# 一种由大体积恶性胸腔积液中分离肿瘤细胞的方法初探

**DOI:** 10.3779/j.issn.1009-3419.2020.103.18

**Published:** 2020-12-20

**Authors:** 闫飞 王, 震 梁, 勇 刘, 芙蓉 寇, 丹凤 姜, 艳群 郑, 巍 刘, 步东 朱

**Affiliations:** 1 100142 北京，北京大学肿瘤医院暨北京市肿瘤防治研究所，恶性肿瘤发病机制及转化研究教育部重点实验室，日间病房 Daycare Center, Key Laboratory of Carcinogenesis and Translational Research (Ministry of Education/Beijing), Peking University Cancer Hospital and Institute, Beijing 100142, China; 2 100142 北京，北京大学肿瘤医院暨北京市肿瘤防治研究所，恶性肿瘤发病机制及转化研究教育部重点实验室，胸外一科 Department of Thoracic Surgery I, Key Laboratory of Carcinogenesis and Translational Research (Ministry of Education/Beijing), Peking University Cancer Hospital and Institute, Beijing 100142, China; 3 102206 北京，康为世纪生物科技有限公司 ComWin Biotech Co., Ltd, Beijing 102206, China

**Keywords:** 胸腔积液, 分离肿瘤细胞, 细胞分离介质, 基因突变检测, 二代测序, Pleural effusion, Tumor cells isolation, Cell separation media, Gene mutation detection, Next-generation sequencing

## Abstract

**背景与目的:**

恶性胸腔积液（malignant plural effusion, MPE）是液体活检基因检测的常用标本来源，本研究评估一种从大体积MPE中分离肿瘤细胞方法的分离效果及其在基因检测中的应用前景。

**方法:**

收集20例伴MPE的晚期肺癌患者一次胸腔积液引流的全部MPE（> 500 mL），联合运用细胞分离介质Percoll和Ficoll分离肿瘤细胞，统计分离情况。对其中既往行组织基因检测的肺腺癌患者，分别使用胸腔积液上清游离DNA（tumor derived DNA from pleural effusion supernatant, etDNA），总细胞DNA及分离肿瘤细胞DNA（DNA from tumor cells in pleural effusion, ETC-DNA）3种成分进行二代基因测序，比较检测情况。

**结果:**

从MPE中分离得到细胞中位数量8.50×10^4^个（上下四分位间距9.25×10^3^-3.75×10^5^），肿瘤细胞纯度85.50%±5.80%。对10例既往行组织表皮生长因子受体（epidermal growth factor receptor, *EGFR*）基因检测的患者，etDNA、总细胞DNA及ETC-DNA *EGFR*基因突变检出率分别为70.00%、50.00%、70.00%。ETC-DNA基因检测阳性率与组织（*P* > 0.999, kappa=1.000）和etDNA（*P* > 0.999, kappa=1.000）一致性均良好。etDNA、总细胞DNA、ETC-DNA *EGFR*基因突变中位丰度分别为16.05%（4.78%-43.06%）、1.09%（0.00%-2.39%）和33.02%（18.50%-76.70%）。ETC-DNA检测丰度倾向高于etDNA，但差异无统计学意义。

**结论:**

该提取方法可以有效地从大体积MPE中分离出大量高纯度肿瘤细胞，利用提取出的肿瘤细胞进行基因检测可能可以提高基因检测效能，值得进一步研究。

## Abstract (en)

**Background and objective:**

Malignant plural effusion (MPE) is one of the most common specimen for liquid biopsy gene detection. This study aims to explore a method for isolating tumor cells from large volume of MPE and evaluate its efficacy and application prospect in gene detection.

**Methods:**

Pleural effusions (> 500 mL) from 20 advanced lung cancer patients were obtained by effusion drainage and used to isolate tumor cells with cell separation media Percoll and Ficoll. Cell number and purity were calculated. DNA was extracted from the supernatant (etDNA), total cells and isolated tumor cells of pleural effusion (ETC-DNA) to detect the mutation of tumor-related genes by next-generation sequencing.

**Results:**

The median number of cells isolated from malignant pleural effusion was 8.50×10^4^ (interquel range: 9.25×10^3^-3.75×10^5^), 85.50%±5.80% of the cells were identified as tumor cells. The detection rates of epidermal growth factor receptor (*EGFR*) gene mutation of etDNA, total cell DNA and ETC-DNA were 70.00%, 50.00% and 70.00%, reseparately, while the median *EGFR* mutation abundance in 3 components was 16.05% (4.78%-43.06%), 1.09% (0.00%-2.39%), and 33.02% (18.50%-76.70%), respectively. ETC-DNA had good consistency with tissue DNA (*P* > 0.999, kappa=1.000) and etDNA (*P* > 0.999, kappa=1.000). ETC-DNA inclined to have higher *EGFR* mutation than etDNA, but the result was not statistically significant.

**Conclusion:**

Our method can isolate large amount of tumor cells from a large volume of malignant pleural effusion with high purity. Using ETC-DNA as specimen improves the efficacy of gene detection, thus is worth further study.

恶性胸腔积液（malignant pleural effusion, MPE）是晚期恶性肿瘤常见并发症，如非小细胞肺癌（non-small cell lung cancer, NSCLC）、乳腺癌、淋巴瘤等^[1]^。分离和鉴定MPE中肿瘤细胞及其基因突变类型对于疾病诊断、病情评估以及治疗策略的制定有重要临床意义。目前，有多种方法用于从MPE中分离肿瘤细胞，如激光捕获显微切割技术、流式细胞分选技术以及磁珠捕获等，但这些方法共同缺点是不能实现从大体积MPE中高通量分选肿瘤细胞，部分技术在分选过程中还需对细胞进行固定和染色，使得分离细胞不能进行后续培养和分析，进一步限制了这些方法的广泛运用^[2-4]^。

本研究基于肿瘤细胞与正常细胞在细胞分离液中沉降系数不同，联合运用细胞分离介质Percoll和Ficoll通过密度梯度离心方法分离、富集大体积MPE中的肿瘤细胞。

## 1 材料与方法

### 1.1 研究对象

选取2018年8月-2018年10月于北京大学肿瘤医院日间病房门诊操作室进行胸腔积液引流的20例连续的伴MPE的晚期肺癌患者，留取一次引流操作收集的全部胸腔积液，胸腔积液量500 mL-1, 000 mL。患者平均年龄（60.9±11.4）岁，男性13例，女性7例，男女比例1:0.54。所有患者均经病理确诊为肺癌，其中腺癌18例，小细胞癌2例。其中10例肺腺癌患者已进行肿瘤组织二代基因测序（next-generation sequencing, NGS）基因检测，检测实验室与panel不限。所有患者胸腔积液脱落细胞学均找见肿瘤细胞。本研究经医学伦理委员会批准，所有患者均签署了知情同意书。

### 1.2 试剂

Percoll细胞分离液（上海前尘生物科技有限公司），Ficoll-Paque^TM^ PLUS单核细胞分离液（GE Healthcare），RPMI-1640培养基（Thermo Fisher）。

### 1.3 研究方法

#### 1.3.1 胸腔积液肿瘤细胞分离

分离方法在既往文献^[5]^基础上进行适量调整。将患者一次胸腔积液引流收集所有胸腔积液2, 000 rpm室温离心10 min。留取10 mL上清用作提取游离DNA，弃多余上清后用RPMI-1640培养基重悬细胞，吸取部分总细胞以提取基因组DNA，调整细胞浓度为10^7^个/mL。离心管加Ficoll-Paque^TM^ PLUS单核细胞分离液后按1:1体积比缓慢加入细胞悬液，2, 000 rpm室温离心25 min，此时，Ficoll-Paque^TM^ PLUS单核细胞分离液与RPMI-1640培养基交界面细胞为肿瘤细胞与白细胞，红细胞沉于管底。吸取交界面细胞，1, 000 rpm离心5 min后用RPMI-1640培养液重悬细胞，调整细胞浓度为10^7^个/mL。离心管加入45% Percoll后，按1:1体积比再缓慢加入细胞悬液。2, 000 rpm室温离心25 min。此时，Percoll与RPMI-1640培养基交界面细胞为肿瘤细胞，离心管底部细胞为以淋巴细胞为主白细胞。

#### 1.3.2 细胞鉴定

将分离所得肿瘤细胞送赛特生物医药科技有限公司进行免疫荧光染色与染色体荧光原位杂交（immunostaining-fluorescence *in situ* hybridization, iFISH）检测，采用抗白细胞分化抗原45（CD45）和白细胞分化抗原31（CD31）的抗体以及8号染色体着丝粒（CEP8）探针，鉴定分离细胞是否为肿瘤细胞，并计算肿瘤细胞的纯度。肿瘤细胞判定标准：DAPI（细胞核标记）阳性; CD45阴性; CD31阴性; CEP8显示异倍体。荧光显微镜下随机选取20个视野，肿瘤细胞的纯度=[DAPI（+）CD45（-）CD31（-）CEP8（异倍体）细胞数]/DAPI（+）细胞数×100%。

#### 1.3.3 DNA提取

分别对胸腔积液上清、胸腔积液总细胞及分离肿瘤细胞（tumor cells in pleural effusion, ETCs）进行DNA提取。其中胸腔积液上清游离DNA（tumor derived DNA from pleural effusion supernatant, etDNA）采用康为世纪的游离DNA提取试剂盒CW2612提取; 胸腔积液总细胞DNA及ETC-DNA采用康为世纪的血液基因组DNA提取试剂盒CW2087提取; 提取方法无调整。

#### 1.3.4 基因检测

将所提取的etDNA、胸腔积液总细胞DNA及ETC-DNA送江苏康为世纪生物科技有限公司应用NGS技术行基因检测（cancer50）。

### 1.4 统计学方法

使用SPSS 16.0软件进行统计学分析。符合正态分布的连续型变量使用均数±标准差表示; 不符合正态分布的连续型变量使用中位数及上下四分位间距表示; 分类数据比较使用*Kappa*检验，连续型变量比较使用*Wilcoxon*符号秩检验。以*P* < 0.05为差异有统计学意义。

## 2 结果

### 2.1 MPE肿瘤细胞分离情况

ETCs形态学特征如图 1所示，可见成簇中大型细胞，出现细胞质空泡化，细胞核形态不一。经计数，从胸腔积液中分离得到肿瘤细胞中位数量8.50×10^4^（上下四分位间距9.25×10^3^-3.75×10^5^）个。经iFISH检测显示DAPI（+）CD45（-）CD31（-）CEP8（异倍体），提取细胞符合肿瘤细胞特征性标记（图 2）。随机选取20个视野，肿瘤细胞的纯度85.50%±5.80%。

**图 1:**
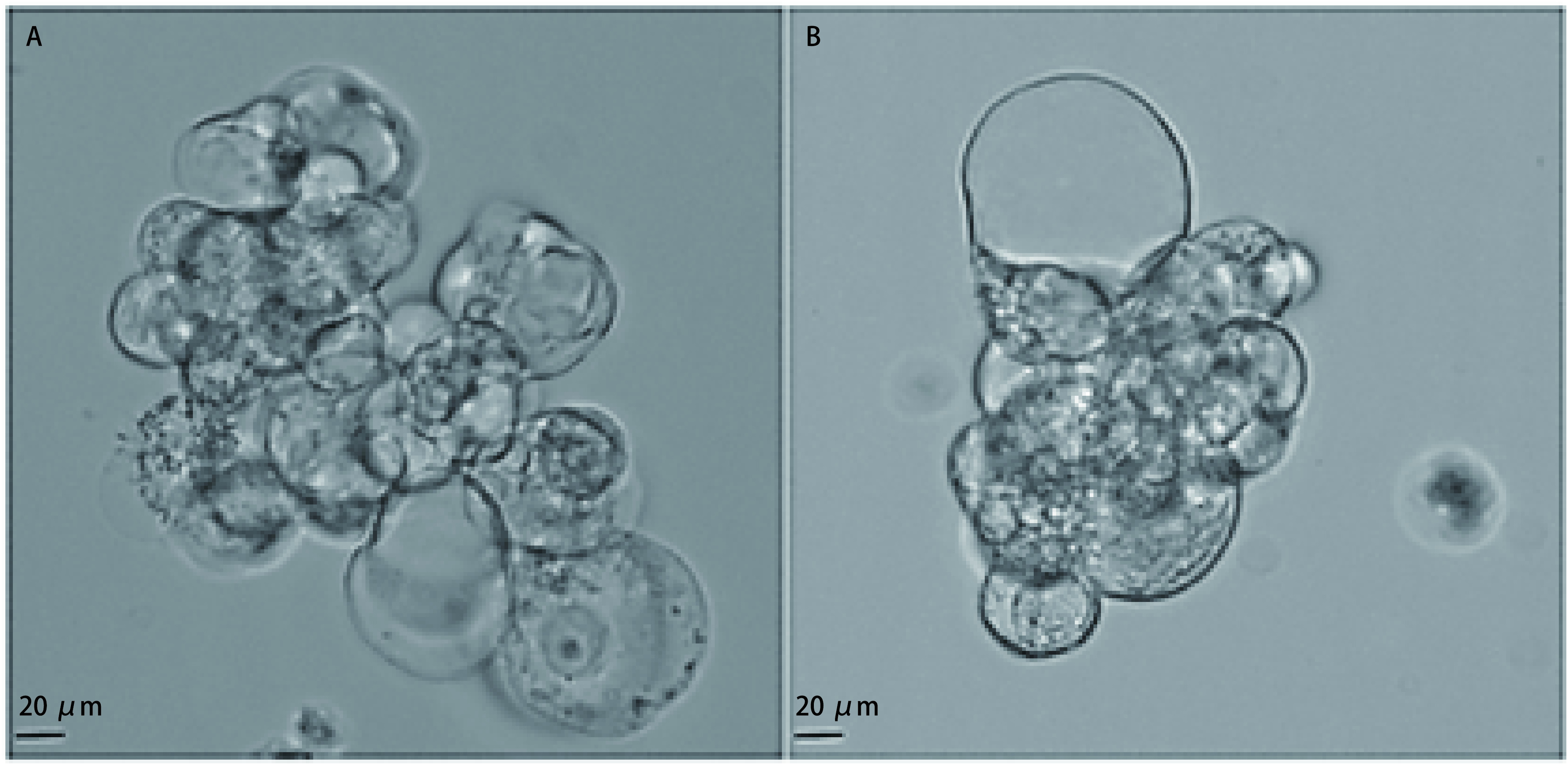
恶性胸腔积液分离肿瘤细胞形态学特征。A、B可见成簇中大型细胞，出现细胞质空泡化，细胞核形态不一。 Morphological characteristics of tumor cells isolated from malignant pleural effusion. There are clusters of large cells with cytoplasmic vacuolization and heterogeneous nuclei (A, B).

**图 2:**
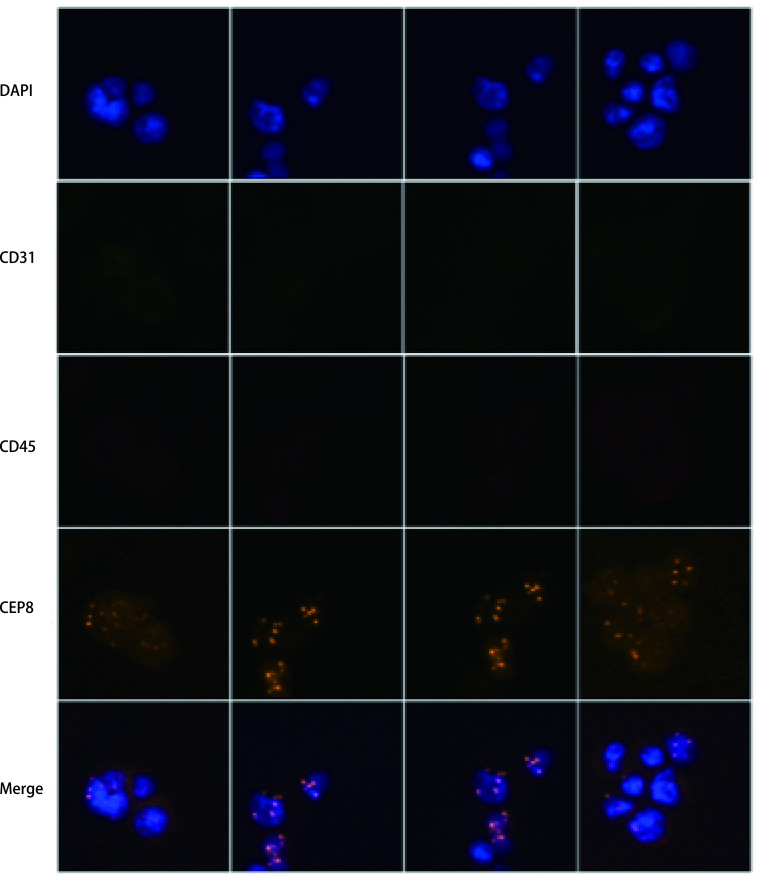
恶性胸腔积液分离肿瘤细胞iFISH检测。应用iFISH检测鉴定分离肿瘤细胞肿瘤细胞判定标准：DAPI（细胞核标记）阳性；CD45阴性；CD31阴性；CEP8显示异倍体。 Identifying isolated tumor cells by iFISH. Criteria for tumor cells: DAPI (nuclear marker) positive; CD45 negative; CD31 negative; CEP8 shows aneuploid. iFISH: immunostaining-fluorescence *in situ* hybridization; DAPI: 4', 6-diamidino-2- phenylindole.

### 2.2 比较10例肺腺癌患者MPE不同分离组分基因突变检测情况

10例行肿瘤组织NGS基因检测的肺腺癌患者中，组织表皮生长因子受体（epidermal growth factor receptor, *EGFR*）基因突变检出率为70.00%，其中6例患者为外显子21 L858R突变，1例为外显子19 L747-A750 > P缺失突变（图 3）。对于该10例患者，使用NGS技术对etDNA、总细胞DNA及ETC-DNA进行基因突变检测，*EGFR*基因突变检出率分别为70.00%、50.00%和70.00%（图 3，表 1）。与使用肿瘤组织进行基因检测检测金标准相比，检测结果一致性分别为100.00%（*P* > 0.999, kappa=1.000）、80.00%（*P*=0.500, kappa=0.600）和100.00%（*P* > 0.999, kappa=1.000）。ETC-DNA与etDNA相比，一致性较好（*P*=1.000, kappa=1.000）（图 4）。

**图 3:**
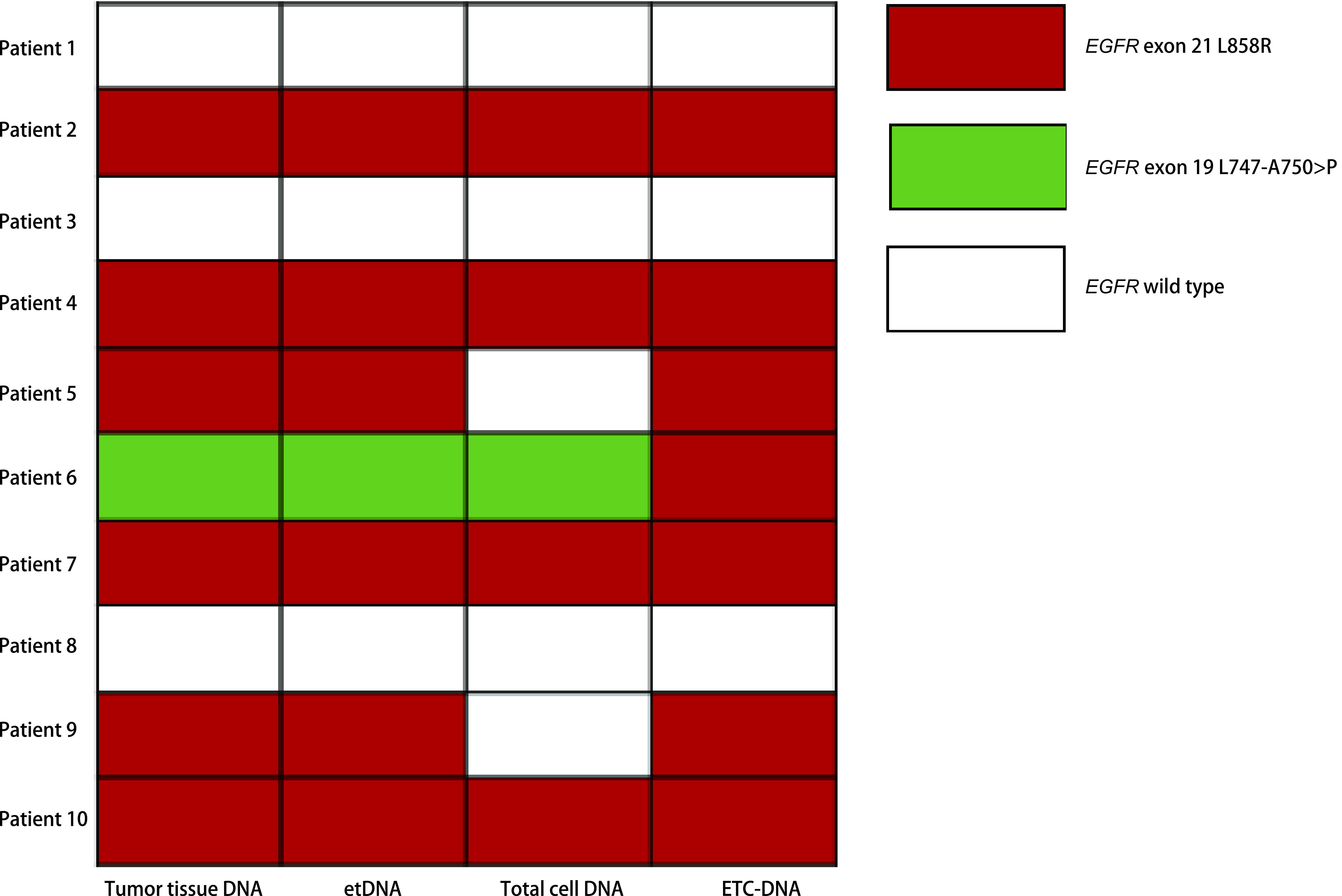
10例肺腺癌患者肿瘤组织及胸腔积液不同组分*EGFR*基因检出情况 *EGFR* mutation status in tumor tissue and different components of pleural effusion in 10 lung adenocarcinoma patients

**图 4:**
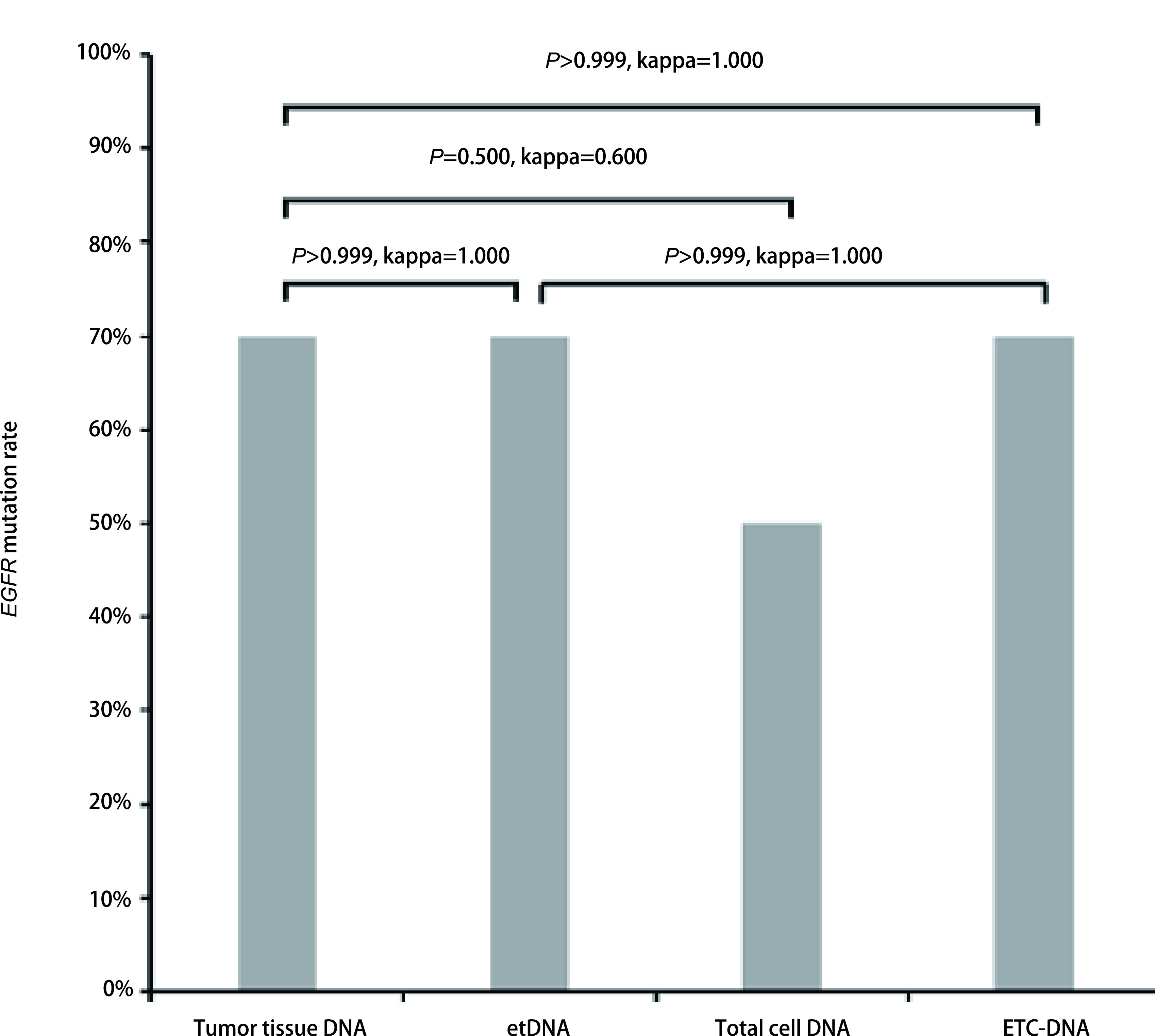
10例肺腺癌患者肿瘤组织及胸腔积液不同组分*EGFR*基因检出率 *EGFR* mutation rates in tumor tissue and different components of pleural effusion in 10 lung adenocarcinoma patients

对于3种组分中至少一种*EGFR*突变检测结果为阳性的7例患者，etDNA、总细胞DNA、ETC-DNA基因突变中位丰度分别为16.05%（4.78%-43.06%）、1.09%（0.00%-2.39%）和33.02%（18.50%-76.70%）（表 1，图 5）。ETC-DNA检测丰度倾向于高于etDNA检测丰度，但差异无统计学意义（*P*=0.310）; 两者均高于总细胞检测丰度（*P*=0.018; *P*=0.018）。

**表 1:** 肺腺癌患者*EGFR*基因检测情况 *EGFR* mutation status in lung adenocarcinoma patients

Item	etDNA	Total cell DNA	ETC-DNA
*EGFR* mutation rate (*n*=10)	70.00%	50.00%	70.00%
*EGFR*mutation abundance [median, (interquel range)] (*n*=7)	16.05% (4.78%-43.06%)	1.09% (0.00%-2.39%)	33.02% (18.50%-76.70%)
EGFR: epidermal growth factor receptor; etDNA: tumor derived DNA from pleural effusion supernatant; ETC-DNA: DNA from tumor cells in pleural effusion.			

**图 5:**
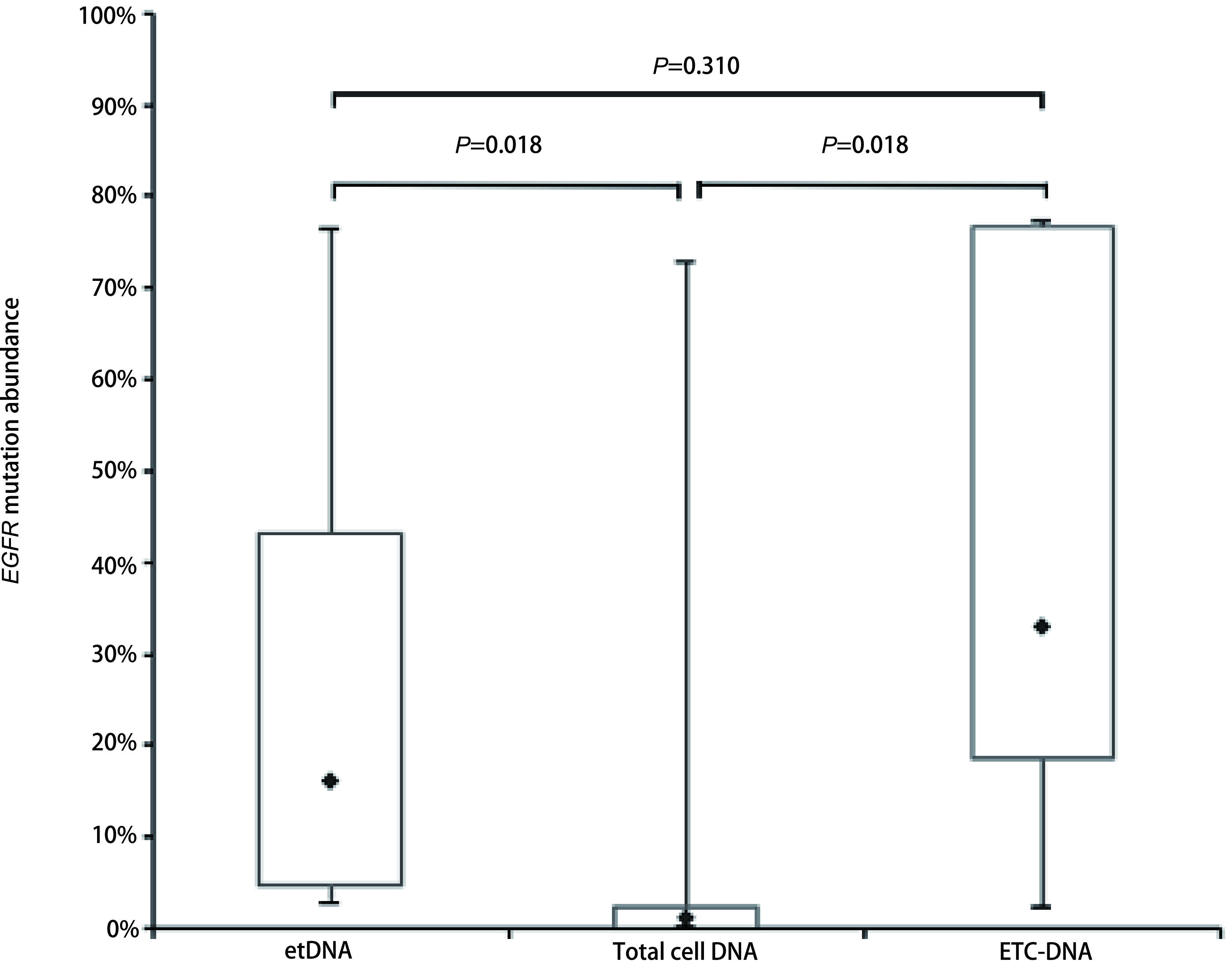
7例肺腺癌患者胸腔积液不同组分*EGFR*基因突变丰度 *EGFR* mutation abundance in different components of pleural effusion in 7 lung adenocarcinoma patients

## 3 讨论

### 3.1 MPE中肿瘤细胞提取

MPE中细胞成分以红细胞、淋巴细胞为主，肿瘤细胞相对较少。在已有的分离细胞方法中，激光捕获显微切割技术所获得的肿瘤细胞纯度最高，认为是金标准，但这项技术获得细胞数量较少，且过程中需干燥细胞，导致细胞损伤，无法进行后续分析^[6]^。流式细胞分选技术是分离细胞的常用方法，处理效率较激光捕获显微切割技术更高，但由于需固定细胞和进行细胞类型特异性抗体标记，使得分选细胞不能用于活细胞实验和继续扩增培养^[6]^。磁珠捕获也是基于细胞类型特异性抗体分选技术，但受处理量限制，也仅能获得较少量的目标细胞^[4]^。本研究采用常见细胞分离介质Percoll和Ficoll联合分离肿瘤细胞，分离过程中无需特殊设备和特异性抗体，成本相对低廉，实现了从大体积MPE中大量分离肿瘤细胞，可获得10^4^左右的肿瘤细胞，iFISH检测纯度达到80%以上，提取质量稳定，具有较高的应用前景。

### 3.2 MPE中分离肿瘤细胞用于液体活检的前景

NSCLC *EGFR*基因突变情况与酪氨酸激酶抑制剂临床效果密切相关^[7]^，使*EGFR*基因突变检测有重要临床意义。肿瘤组织被认为是进行基因检测金标准，然而很多患者因活检困难等原因难以获得肿瘤组织，使得液体活检成为基因检测优秀替代。血液循环肿瘤DNA（circulatory tumor DNA, ctDNA）是常用液体活检来源，但由于ctDNA含量少，检测敏感率低^[8]^。MPE是晚期NSCLC的常见并发症，发生率高达60%^[1]^，标本易得，取材安全，其中存在大量脱落的恶性肿瘤细胞及肿瘤细胞破碎而来的etDNA，可以体现实时的肿瘤突变状态，检测效果优于ctDNA^[9]^，成为基因检测的新领域^[8]^。传统的研究使用MPE沉渣细胞制成的涂片或石蜡包埋标本进行检测。MPE沉渣由红细胞、淋巴细胞为主的白细胞和肿瘤细胞构成，因其中混入了大量正常组织细胞，影响了检测效能，导致检测敏感性差。Tong等^[9]^研究采用MPE离心后的上清提取的etDNA进行二代测序检测，发现etDNA肿瘤特有突变检出率98%，高于胸水沉渣细胞DNA（89%）和外周血ctDNA（86%），但仍差于肿瘤组织（检出率为100%）; 在肺癌*EGFR*突变检出率中，胸水上清检出率为71%，高于胸水沉渣细胞（68%）和外周血（59%），而Zhou等^[10]^的研究显示，胸水上清etDNA所检出突变的丰度高于胸水沉渣DNA和ctDNA，与肿瘤组织相近。尽管etDNA的使用已经极大改善了液体活检的检测敏感性，然而，由于胸腔积液中细胞成分复杂，胸腔积液上清中同样混有大量正常细胞坏死而来的DNA片段，会干扰对目标基因突变状态的检测，导致检测敏感性低于肿瘤组织。对于上清液中DNA片段的混杂，现有手段无法进行分离; 而采用合适的分离技术纯化肿瘤细胞，优化标本质量，从而去除MPE沉渣细胞中正常细胞基因组对测序信息的掩盖，为理论可行。Wang等^[11]^通过使用流式细胞学手段富集MPE中恶性肿瘤细胞以提高应用等位基因特异的TaqMan聚合酶链式反应（competitive allele-specific TaqMan polymerase chain reaction, CAST-PCR）手段对NSCLC患者*EGFR*基因检测的效能，该研究显示，28例NSCLC患者的MPE标本中，应用流式细胞学手段对MPE中的恶性肿瘤细胞进行富集之后，肿瘤细胞中位浓度由0.64%升至40.8%，*EGFR*基因突变检测阳性率由28.6%提升到42.9%，检测敏感性提高了44.4%，提示通过对MPE中肿瘤细胞的提取、富集是提高基因检测效能的可行方式，但该研究应用流式细胞学对肿瘤细胞进行提取，技术相对复杂，费用昂贵，限制了该方法的推广应用。

我们联合用两种常用细胞提取介质，对肿瘤细胞进行了有效提取。使用NGS基因检测发现，在MPE沉渣中去除体内正常细胞后，ETC-DNA *EGFR*突变检出率提高，与组织检出率一致性良好，中位突变丰度明显增加，有高于etDNA突变丰度的趋势。进一步改善提纯技术并扩大样本量后，ETC-DNA的检测效率有可能进一步提高，优于etDNA，有待后续研究证实。

综上，本研究建立了一种从晚期肿瘤患者MPE中分离肿瘤细胞方法，与其他分离方法相比，该方法可实现从大体积MPE中分离得到大量纯度较高肿瘤细胞，且操作简便、价格低廉。在去除正常细胞基因组干扰后，使用分离所得的肿瘤细胞进行基因检测，基因突变检测率与组织DNA一致性良好，检测丰度有高于etDNA的趋势。该方法有较高应用前景，值得推广及进一步研究。
